# ScnML models single-cell transcriptome to predict spinal cord neuronal cell status

**DOI:** 10.3389/fgene.2024.1413484

**Published:** 2024-06-04

**Authors:** Lijia Liu, Yuxuan Huang, Yuan Zheng, Yihan Liao, Siyuan Ma, Qian Wang

**Affiliations:** ^1^ School of Recreation and Community Sport, Capital University of Physical Education and Sports, Beijing, China; ^2^ Department of Neuroscience in the Behavioral Sciences, Duke University and Duke Kunshan University, Suzhou, Jiangsu, China; ^3^ Taizhou Hospital of Zhejiang Province, Wenzhou Medical University, Luqiao, China; ^4^ Department of Neurology, The First Hospital of Tsinghua University, Beijing, China

**Keywords:** machine learning, spinal cord nervous, ScRNA-seq, marker genes, cell subpopulations

## Abstract

Injuries to the spinal cord nervous system often result in permanent loss of sensory, motor, and autonomic functions. Accurately identifying the cellular state of spinal cord nerves is extremely important and could facilitate the development of new therapeutic and rehabilitative strategies. Existing experimental techniques for identifying the development of spinal cord nerves are both labor-intensive and costly. In this study, we developed a machine learning predictor, ScnML, for predicting subpopulations of spinal cord nerve cells as well as identifying marker genes. The prediction performance of ScnML was evaluated on the training dataset with an accuracy of 94.33%. Based on XGBoost, ScnML on the test dataset achieved 94.08% 94.24%, 94.26%, and 94.24% accuracies with precision, recall, and F1-measure scores, respectively. Importantly, ScnML identified new significant genes through model interpretation and biological landscape analysis. ScnML can be a powerful tool for predicting the status of spinal cord neuronal cells, revealing potential specific biomarkers quickly and efficiently, and providing crucial insights for precision medicine and rehabilitation recovery.

## Introduction

The spinal cord nerves are the primary regulators of a wide range of motor behaviors in animals, which cover a range of fine motor actions from basic fight or flight responses to complex social interactions (Liau et al., 2023a). When the spinal nerves are abnormal, the patient quickly enters a phase known as “spinal shock,” which can lead to permanent loss of motor, sensory, and autonomic functions (Li et al., 2022). Spinal cord injury (SCI) is a traumatic neurological disorder, especially lower thoracic and cervical spine lesions causing paraplegia and quadriplegia (Alizadeh et al., 2019). A detailed understanding of spinal cord nerves provides important implications for the future development of more precise clinical treatments or guided exercise training to promote functional recovery after SCI, as well as for the conduct of pathophysiologic research (Fu et al., 2016; Alizadeh et al., 2019).

With the development of single-cell sequencing technique (Xiong et al., 2020; Xiong et al., 2022), we can explore the cellular composition of spinal nerves at high resolution. For example, Liau et al. (2023b) used scRNA sequencing to resolve the heterogeneity of mouse spinal motor neurons and discovered a diverse code of neuropeptide to characterize putative motor pool identities. Based on single-cell RNA sequencing (scRNA-seq) technique, Wang T. et al. (2023) resolved the cellular heterogeneity of orthopedic diseases, including spinal cord injury (SCI), related to their development, as well as their functions and potential molecular mechanisms. Cao et al. (2022) utilized single-cell RNA sequencing (scRNA-seq) to comprehensively depict the cellular diversity of the spinal cord, deeply reveal the dynamic changes of cells and molecules in the microenvironment, and elucidate the intercellular communication between the normal and injured states of the spinal cord, which provides a powerful tool for the study of the molecular mechanisms of traumatic spinal cord injury. Delile et al. (2019) used single-cell mRNA sequencing to resolve developmental maps of the cervical and thoracic regions of the neural tube in mice on embryonic days 9.5–13.5, revealing mechanisms of neuronal specification and providing direct insights into spinal cord cell classification.

Despite the fact that previous research techniques are quite mature, there are time-consuming and laborious problems in mining marker genes and identifying cell subpopulations using manual methods. Therefore, there is an urgent need to develop a computational method to assist researchers in efficiently identifying cellular subpopulations and deeply exploring their potential marker genes.

**FIGURE 1:**
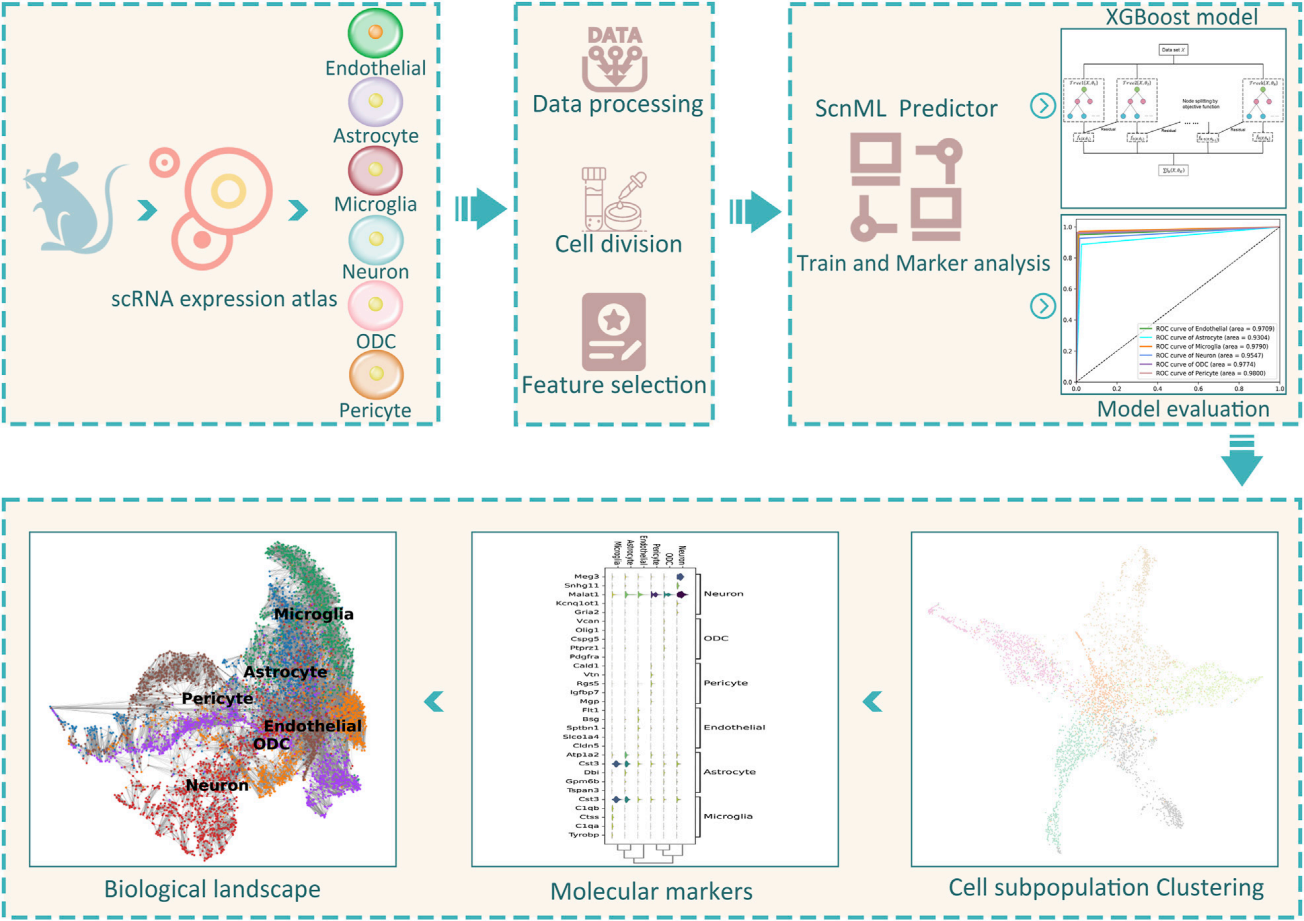
The workflow of constructing ScnML.

To overcome these challenges, we introduced a computational framework, called ScnML, designed to identify biomarkers of cell subpopulations within the spinal cord neuronal and to predict cellular developmental stages. The framework is shown in Figure 1. In order to obtain the optimal predictive model, we used a strategy that combines feature selection and incremental feature selection (IFS) (Wang et al., 2021a) in four basic classification methods: K-Nearest Neighbors (KNN), extreme Gradient Boosting (XGBoost) (Chen and Guestrin, 2016), Support Vector Machine (SVM) (Cortes and Vapnik, 1995; Zhang et al., 2024), and Random Forest (RF) (Al-Allak et al., 2013). Given the importance of interpretability and robustness, we chose the XGBoost algorithm to build the computational model. We validated the model using a test set and achieved an accuracy of 94.04%. By performing biological analysis of the optimal genes, we identified potential marker genes that may assist biologists in gaining a deeper insight into the diversity present within spinal cord neuronal.

### Identification of significant genes by ScnML

**FIGURE 2:**
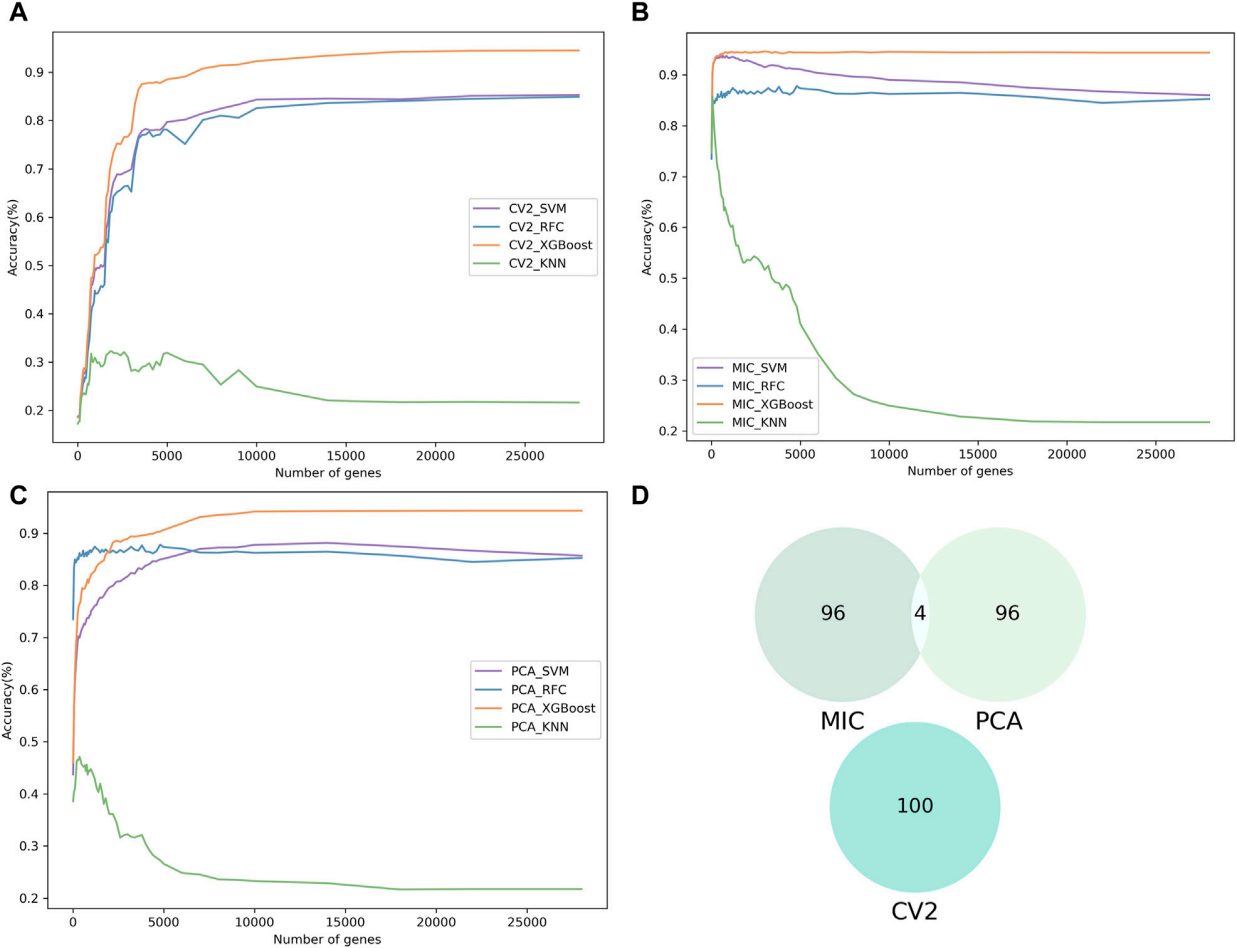
The results of feature selection. **(A–C)** Show the incremental feature selection (IFS) curves illustrating the prediction performance of the three feature selection methods (CV2, MIC and PCA) with four different classifiers for different gene subsets. **(D)** Comparative Venn diagram of the top 100 genes in MIC, CV2 and PCA.

To identify significant genes associated with spinal cord neuronal cell subpopulations, we used three feature selection methods (Mutual Information Coefficient: MIC, Coefficient of Variation Squared: CV2, and Principal Component Analysis: PCA) to assess the significance of 27,998 genes and ranked them according to their contribution values. Genes with importance scores less than or equal to zero were excluded. Next, the machine learning models were combined with IFS to determine the optimal subset of genes (Figures 2A–C). Machine learning models (SVM, RFC, XGBoost, and KNN) were trained using single-cell gene expression matrices (Normalized of raw read count) as input features, based on five-fold cross-validation.

The results from the training dataset showed that MIC combination with XGBoost (ScnML), achieved the optimal prediction performance by using the first 210 genes, with an accuracy of 94.33% (Table 1). Based on the 210 best genes, ScnML also achieved the best performance on the test dataset, with accuracy, precision, recall, and F1_metrics of 94.08%, 94.24%, 94.26%, and 94.24%, respectively (Table 2). It is notable that the four machine learning models, when combined with PCA, also yielded superior predictive performance. To avoid feature selection methods having the same scoring preferences, we compared the top 100 genes scored by the three feature selection methods. As observed from Figure 2D, there is almost no intersection among the top 100 genes selected by MIC, CV2, and PCA, demonstrating the effectiveness of each feature selection method.

**TABLE 1:** Performance evaluation of different feature selection combined with machine learning schemes (Train dataset).

Method	Feature selection	No. of features	Accuracy (%)
KNN	PCA	360	47.15
XGBoost	PCA	10,000	94.11
SVM	PCA	14,000	88.14
RFC	PCA	4,800	87.79
KNN	CV2	760	31.72
XGBoost	CV2	10,000	92.29
SVM	CV2	10,000	84.33
RFC	CV2	18,000	87.04
KNN	MIC	60	85.77
XGBoost	MIC	210	94.33
SVM	MIC	660	93.72
RFC	MIC	4,800	87.79

**TABLE 2:** Performance evaluation of different feature selection combined with machine learning schemes (Test dataset).

Method	Feature selection	No. of features	Accuracy (%)	Precision (%)	Recall (%)	F1-measure (%)
KNN	PCA	360	45.23	45.64	46.06	45.47
XGBoost	PCA	10,000	92.89	92.13	92.04	92.33
SVM	PCA	14,000	87.05	87.50	86.61	86.92
RFC	PCA	4,800	87.23	87.36	86.97	87.23
KNN	CV2	760	27.41	27.23	27.02	26.51
XGBoost	CV2	10,000	91.83	92.12	92.04	92.00
SVM	CV2	10,000	82.41	84.35	82.57	82.90
RFC	CV2	18,000	86.33	87.86	86.68	86.85
KNN	MIC	60	86.91	88.64	87.06	87.47
XGBoost	MIC	210	94.08	94.24	94.26	94.24
SVM	MIC	660	93.51	93.80	93.74	93.74
RFC	MIC	4,800	87.13	88.31	87.46	87.62

### Performance of ScnML on the test dataset

**FIGURE 3:**
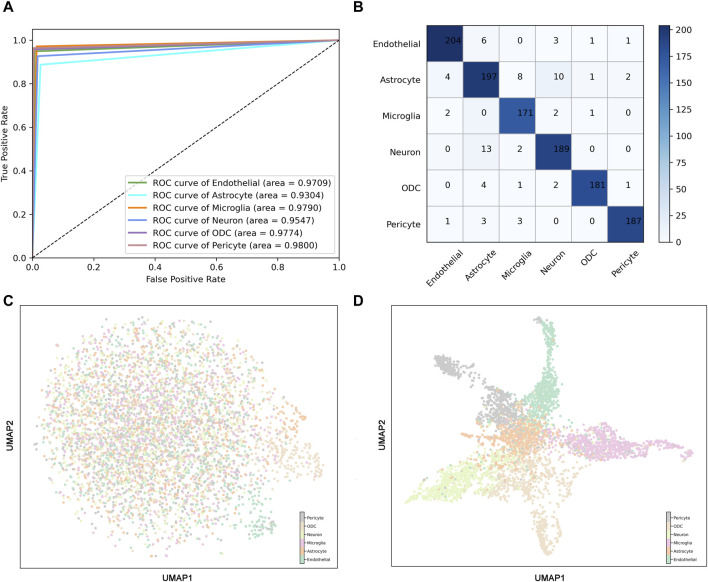
Predictive performance of ScnML. **(A)** The Receiver Operating Characteristic (ROC) curves for the ScnML model evaluated on the training dataset. **(B)** Confusion matrix for ScnML, used to assess the predictive performance of the model for each cell subpopulation classification. **(C)** UMAP shows clustering performance for six spinal cord nervous cell subpopulations at all gene set levels. **(D)** UMAP shows clustering performance for six spinal cord nervous cell subpopulations at ScnML gene set levels.

To further validate the robustness of the model, receiver operating characteristic (ROC) curves and confusion matrices were used to evaluate the prediction performance of ScnML. We observe that the AUC of the ScnML model is 0.96 (Figure 3A). The confusion matrix validates the predictive performance of the model for each type of spinal cord neural subpopulation, and the low misclassification rate demonstrates the robustness of the model (Figure 3B). In addition, Uniform Manifold Approximation and Projection (UMAP) of 6,000 single cells revealed that the overall performance of the 210 marker genes was significantly better than that of all genes (Figures 3C, D). In particular, the samples from different categories were almost blended together in the clustering process that exploited all genes (Figure 3C). However, employing the 210 optimal genes generates a distinct distribution of cell subpopulations, demonstrating clear clustering findings (Figure 3D). We also performed heat map clustering analysis on the ScnML gene set and obtained excellent clustering results, demonstrating the advantages of machine learning (Supplementary Figure S1).

### Gene function analysis

We performed functional enrichment analysis of the ScnML gene set to explore biological processes related to sci pathophysiology and potential recovery mechanisms. The analysis revealed significant enrichment in genes associated with axon ensheathment, myelination, and the ensheathment of neurons, highlighting the pivotal role of myelin repair and axonal regeneration post-injury (Franklin and Ffrench-Constant, 2008; Lee et al., 2012) (Supplementary Figure S2). Additionally, processes such as glial cell differentiation and gliogenesis were prominently featured, underscoring the importance of glial responses in scar formation and neural tissue remodeling (Sofroniew, 2009). Importantly, our findings also suggest that the regulation of cell-substrate adhesion and leukocyte migration, including myeloid cells, as key components in the inflammatory response and subsequent healing processes (Supplementary Figures S3, S4). The modulation of cell adhesion dynamics is particularly critical, as it influences axonal growth and neural cell interaction with the extracellular matrix, which are essential for effective nerve repair (Zhu et al., 2015).

### Expression analysis of the ScnML gene set

**FIGURE 4:**
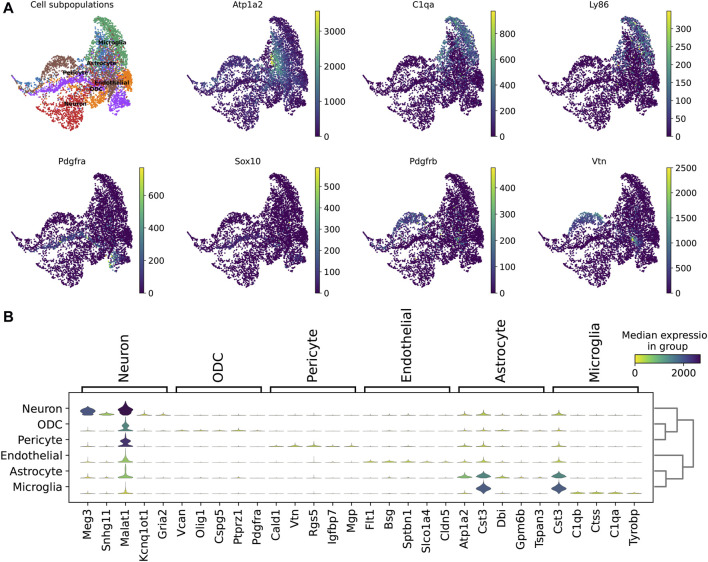
Computational analysis of ScnML gene set. **(A)** UMAP shows reported marker genes for spinal cord neuronal cell subpopulations. **(B)** Violin plot shows potential marker genes for subpopulations of spinal cord nerve cells.

In addition, we explored the representation of the 210 marker genes in the biological landscape. We identified potential marker genes such as Atp1a2, which is highly expressed in astrocytes; C1qa and Ly86, which are specifically expressed in microglia; and Vtn, which characterizes a subpopulation of endothelial cells (Figure 4A). These genes have been verifiably reported. Furthermore, the use of multiple genes to characterize cellular subpopulations improves accuracy. For instance, Meg3, Snhg11, and Malat1 ensure the identification of neuron subpopulations; Atp1a2, Cst3, and Dbi are highly expressed in astrocytes; and Cst3, C1qb, and Ctss exhibit high expression levels in microglia (Figure 4B).

**FIGURE 5:**
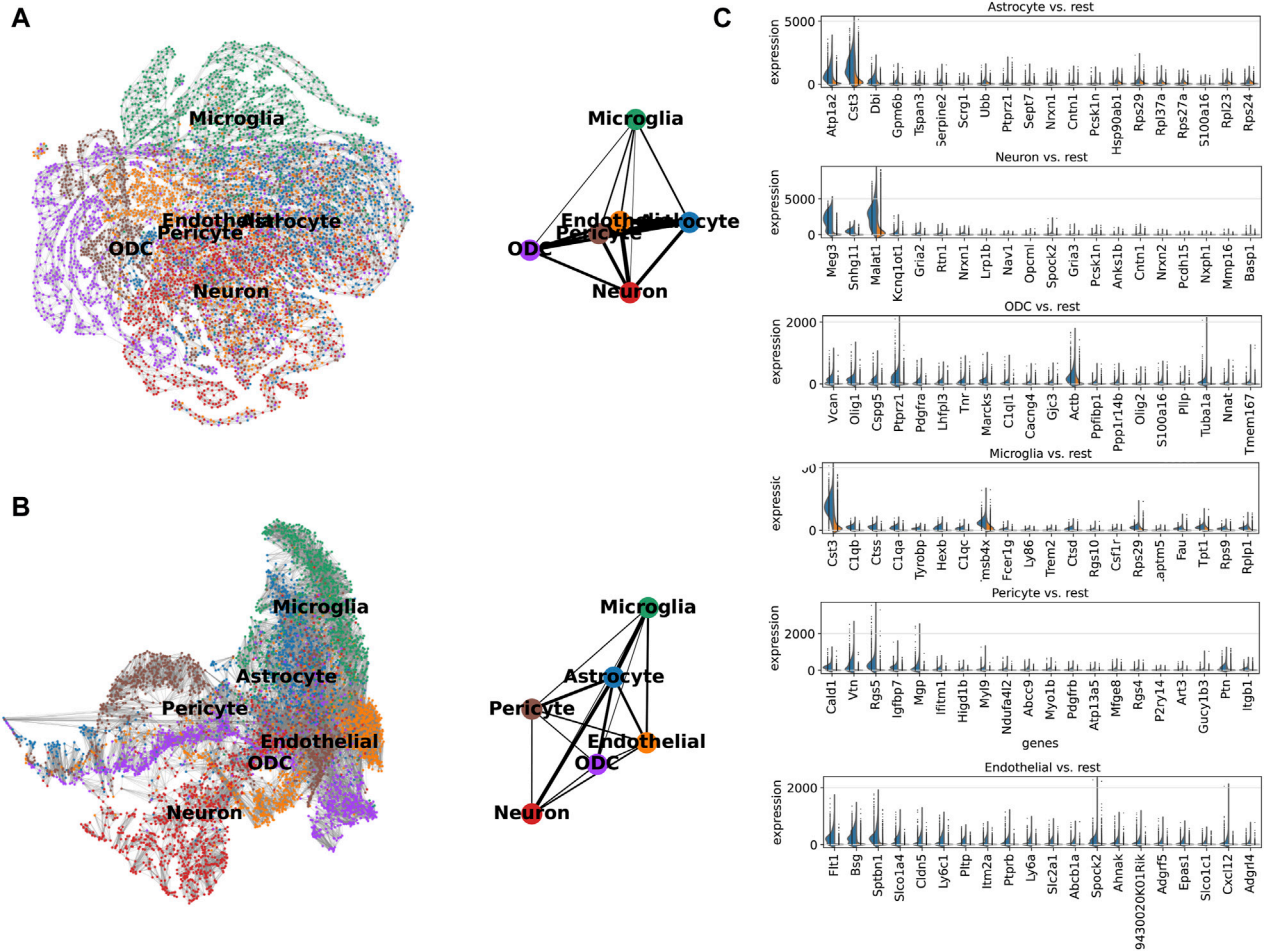
**(A, B)** Expression trajectory analysis of 210 marker genes (downward) and all genes (upward) of spinal cord nerve cell subpopulations colored by cell type using PAGA. The thicker the line, the closer the cell connection. **(C)** Comparison of marker genes selected by ScnML (210 marker genes) using split violin plots. The expression level of marker genes in specific cells is shown on the left (Blue), and the total expression level in the remaining five cell types is shown on the right (Orange).

We analyzed single-cell expression profiles containing all genes and separately, the 210 genes, as the basis for constructing a partition-based graph abstraction (PAGA) to describe the spinal cord neuronal cell bioscape. Both displayed the same topological structure, such as a tight association between microglia and astrocytes, indicating that ScnML screened for key molecular markers and removed redundant information (Figures 5A, B). We utilized Scanpy to compare the expression levels of the top 20 genes in each cell subpopulation with their expression levels in the other five clusters. For example, the expression levels of Cst3, Dbi, and Malat1 in the astrocyte subpopulation were each higher than the combined totals from the remaining five cellular subpopulations. In the neuron cell subpopulation, Meg3, Snhg11, and Malat1 showed high levels of expression, suggesting their potential as marker genes (Figure 5C and Supplementary Table S1). These results indicate that ScnML possesses irreplaceable advantages in processing scRNA-seq data without relying on prior biological knowledge.

## Conclusion

Single-cell sequencing technology has been extensively used in both basic science research and the clinical setting, promoting the exploration of cellular differentiation and molecular heterogeneity. In this research, we designed and developed a machine learning-based predictive model, ScnML, for predicting spinal cord nerve cell subpopulations. ScnML addresses the computational inefficiencies and overfitting problems caused by high-dimensional feature spaces, thereby improving the model’s prediction accuracy and robustness. Results from an independent dataset show that ScnML outperformed other methods, achieving an accuracy of 94.08% and a ROC of 0.96. More significantly, through the analysis of the ScnML model, we have successfully identified a set of key genes that can be utilized as reliable biomarkers for spinal cord neuronal cell subpopulations. This discovery provides an important molecular tool for deeper comprehension of spinal cord nerve cells’ intricacies, with far-reaching impacts on future neurobiology research.

## Methods

### Dataset construction and preprocessing

The single-cell transcriptome dataset of crush-injured adult mouse spinal cord that support the findings of this study are available in figshare with the identifier (https://doi.org/10.6084/m9.figshare.17702045) (Li et al., 2022). Based on the same processing method used by Liu et al. the raw sequence data were aligned to the mm10 (Ensembl 84) reference genome and cell numbers and unique molecular identifiers (UMIs) were estimated using CellRanger (3.1.0). The 6,000 single-cell transcriptome samples were used to classify six spinal cord injury cell subpopulations, including Endothelial, Astrocyte, Microglia, Neuron, Oligodendrocyte (ODC), and Pericyte cells. These single-cell transcriptome samples were randomly divided into a 4800-sample training set and a 2200-sample testing set with a ratio of 7:3. To construct a stringent and robust benchmark dataset, we applied a filtration criterion, excluding genes with unique feature counts of zero or less. This process yielded a final set of 27,998 genes, each expressed in at least one of the 6,000 cells surveyed.

### Mutual information coefficient

The Mutual Information Coefficient (MIC) is predicated on the idea that the presence of a relationship between two variables allows for the construction of a grid that effectively partitions their scatter plot, encapsulating the essence of their interaction. To enable equitable comparisons across grids of different sizes, the mutual information values derived from these partitions are normalized. This normalization ensures a consistent framework for evaluating the strength and complexity of relationships between variables, irrespective of their scale or the intricacy of their association (Zhou et al., 2004; Reshef et al., 2011; Albanese et al., 2012).

$$I\left(X;Y\right)=\sum_{x,y}p\left(x,y\right)\log\frac{p\left(x,y\right)}{p\left(x\right)p\left(y\right)}=H\left(X\right)-H\left(X|Y\right)$$ (1)

Where $I\left(X;Y\right)$ representing Mutual Information Entropy, is a measure of the information about variable $X\;\left(or\;Y\right)$ contained in variable $Y\;\left(or\;X\right)$ .

### Biological analysis

We performed an extensive analysis to assess the represent capability of 210 marker genes in identifying cell subpopulations. The clustering analyses were performed using the Scanpy software (version 1.9.1), and default parameters were used for all analyses (Wolf et al., 2018). Partition-based graph abstraction (PAGA) was also implemented via Scanpy, while uniform manifold approximation and projection (UMAP) visualizations were generated using the umap-learn Python package (version 0.3.9), with parameters set to default values. Furthermore, functional enrichment analysis was executed employing the enrichGO function from the clusterProfiler package (version 4.6.2).

### eXtreme Gradient Boosting

eXtreme Gradient Boosting (XGBoost) is a highly sophisticated and efficient machine learning algorithm that has gained widespread recognition for its performance in various predictive modeling competitions (Chen and Guestrin, 2016; Wang et al., 2023b). XGBoost has gained prominence for its efficiency and effectiveness in various predictive modeling competitions. It operates by constructing a series of decision trees in a sequential manner, where each subsequent tree aims to correct the errors of its predecessors. This approach enables the model to learn complex patterns in the data, enhancing its predictive accuracy. One of the key strengths of XGBoost is its ability to handle large datasets with speed and precision, making it an ideal choice for our study. In addition, compared to models such as KNN and SVM, XGBoost provides a direct way to evaluate the importance of each input variable.

### Model evaluation

The four classic metrics were used to quantify the performance of model predictions, including Accuracy, Recall, Precision, and F1_measure, defined as (Fu et al., 2019; Wang et al., 2021b; Joshi et al., 2021; Liang et al., 2021; Wang et al., 2023c; Liu et al., 2023; Qian et al., 2023):

$$\text{Accuracy}=\frac{TP+TN}{TP+TN+FP+FN}$$ (2)

$$\text{Recall}=\frac{TP}{TP+FN}$$ (3)

$$\text{Precision}=\frac{TP}{TP+FP}$$ (4)

$$F1\text{ measure}=\frac{2*\left(precision*recall\right)}{precision+recall}$$ (5)

Where TP,TN,FP and FN represent the numbers of true positives, true negatives, false positives and false negatives, respectively. In addition, ROC was used to evaluate the performance of the ScnML (Zeng et al., 2016; Zulfiqar et al., 2024).

## Data Availability

The original contributions presented in the study are included in the article/Supplementary Material, further inquiries can be directed to the corresponding authors.
