# Supplementary material for: ScnML models single-cell transcriptome to predict spinal cord neuronal cell status
Source: Front Genet. 2024 Jun 4;15:1413484. doi: 10.3389/fgene.2024.1413484 (PMC11183327; doi:10.3389/fgene.2024.1413484)
Supplement: Supplementary file 1 [file Table5.DOCX]

Table S1. Marker genes and cell states.

| Genes | Cells | Genes | Cells | Genes | Cells |
| --- | --- | --- | --- | --- | --- |
| Rgs5 | Pericyte | 9430020K01Rik | Endothelial | Rtn1 | Neuron |
| Vtn | Pericyte | Rgs4 | Pericyte | Gria2 | Neuron |
| Cald1 | Pericyte | Sptbn1 | Endothelial | Laptm5 | Microglia |
| Vcan | ODC | Cldn5 | Endothelial | P2ry14 | Pericyte |
| C1qb | Microglia | Cacng4 | ODC | Ly6a | Endothelial |
| Ctss | Microglia | Cd9 | ODC | Utrn | Endothelial |
| Flt1 | ODC | Ppfibp1 | ODC | Lbh | ODC |
| C1qa | Microglia | Tpt1 | Endothelial | Tuba1a | ODC |
| Cst3 | Microglia | Myo1b | Pericyte | Gucy1b3 | Pericyte |
| Mgp | Pericyte | Atp13a5 | Pericyte | Kcnj8 | Pericyte |
| Tyrobp | Microglia | Malat1 | Astrocyte | Tsc22d1 | ODC |
| Pdgfra | ODC | Art3 | Pericyte | Eef1a1 | Pericyte |
| Myl9 | Pericyte | Cox4i2 | Pericyte | Xylt1 | ODC |
| Olig1 | ODC | Sulf2 | ODC | 3632451O06Rik | ODC |
| Igfbp7 | Pericyte | Adgrf5 | Endothelial | Rps29 | Pericyte |
| C1ql1 | ODC | Rgs10 | Microglia | Cxcl12 | Endothelial |
| Tmsb4x | Microglia | Trem2 | Microglia | Ifitm3 | Endothelial |
| Meg3 | Neuron | Abcb1a | Endothelial | Mylk | Pericyte |
| C1qc | Microglia | Sema3d | ODC | S100a16 | ODC |
| Hexb | Microglia | Hspb1 | Pericyte | Chst2 | ODC |
| Tnr | ODC | Pltp | Endothelial | Scrg1 | ODC |
| Ifitm1 | Pericyte | Gpr17 | ODC | 3110035E14Rik | ODC |
| Ndufa4l2 | Pericyte | Nbl1 | Pericyte | Ecm2 | Pericyte |
| Cspg4 | ODC | Mfge8 | Pericyte | Rps4x | Pericyte |
| Ptprz1 | ODC | Pllp | ODC | Tm4sf1 | Endothelial |
| Higd1b | Pericyte | Neu4 | ODC | Rplp1 | Pericyte |
| Fcer1g | Microglia | Notch3 | Pericyte | Adgrl4 | Endothelial |
| Lhfpl3 | ODC | Sox10 | ODC | Gm13861 | Pericyte |
| Slco1a4 | Endothelial | Itm2a | Endothelial | Rpl13 | ODC |
| Cspg5 | ODC | Olig2 | ODC | Itga1 | Pericyte |
| Gjc3 | ODC | Actb | ODC | Ccnd1 | ODC |
| Ptprb | Endothelial | Slc38a11 | Pericyte | Rps9 | Pericyte |
| Abcc9 | Pericyte | Dbi | Astrocyte | Epas1 | Endothelial |
| Pdgfrb | Pericyte | Susd5 | ODC | Mmp16 | ODC |
| Ptn | Pericyte | Sept7 | Astrocyte | Sox6 | ODC |
| Snhg11 | Neuron | Arhgap29 | Endothelial | Cntn1 | Neuron |
| Ly86 | Microglia | Nnat | ODC | Mir143hg | Pericyte |
| Ahnak | Endothelial | Csf1r | Microglia | Gucy1a3 | Pericyte |
| Matn4 | ODC | Sept4 | Pericyte | Itgb1 | Pericyte |
| Ly6c1 | Endothelial | Marcks | ODC | Slc2a1 | Endothelial |
| Rpl18a | Microglia | Anks1b | ODC | Nxph1 | ODC |
| Adap2 | Pericyte | Bsg | Endothelial | Tspan3 | Astrocyte |
| Bcas1 | ODC | Rps27a | Pericyte | Rps10 | Astrocyte |
| Fn1 | Endothelial | Pecam1 | Endothelial | Rpl27a | Microglia |
| Fau | Microglia | Itga9 | ODC | Tns1 | Endothelial |
| Ctsd | Microglia | Rpl10 | Pericyte | Rpl30 | Microglia |
| Marcksl1 | ODC | Qpct | ODC | Rplp0 | ODC |
| Ppp1r14b | ODC | Car4 | Endothelial | Esam | Endothelial |
| Crip2 | Endothelial | Rpl35a | ODC | Sod3 | Pericyte |
| Phldb2 | Pericyte | Snx22 | ODC | Kcnq1ot1 | Neuron |
| Gjc1 | Endothelial | Rpl23 | ODC | P2ry12 | Microglia |
| Aif1 | Microglia | Ncald | ODC | Tpm1 | Pericyte |
| Rps15a | Pericyte | Rps20 | Endothelial | Pcsk1n | Neuron |
| Rps24 | Microglia | Wscd1 | ODC | Lrp1b | Neuron |
| Dlc1 | Endothelial | Rps8 | Endothelial | Gria3 | Neuron |
| Basp1 | ODC | Rps16 | Pericyte | Rps14 | Pericyte |
| Slco1c1 | Endothelial | Rpl32 | Pericyte | Siglech | Microglia |
| Rps12 | Microglia | Pglyrp1 | Endothelial | Megf11 | ODC |
| Rps11 | Microglia | Colec12 | Pericyte | Mybpc1 | ODC |
| Pcdh15 | ODC | Opcml | Neuron | Atp1a2 | Astrocyte |
| Arhgap31 | ODC | Pdzd2 | Pericyte | Sirt2 | ODC |
| Gng11 | Pericyte | Tmem167 | ODC | Serpine2 | ODC |
| Rpl39 | Microglia | Rpl37a | Pericyte | Nid1 | Pericyte |
| Perp | Pericyte | Ifitm2 | Pericyte | Kitl | Endothelial |
| Slc6a20a | Pericyte | Rps23 | Pericyte | Sox8 | ODC |
| Hsp90ab1 | Pericyte | Nav1 | Neuron | Rlbp1 | ODC |
| Gpm6b | Pericyte | Spock2 | Neuron | Tagln2 | ODC |
| Rarres2 | Pericyte | Ftl1 | ODC | Ubb | ODC |
| Gper1 | Pericyte | Fxyd6 | ODC |  |  |
| Ace2 | Pericyte | Nrxn1 | Pericyte |  |  |
| Nrxn2 | ODC | Filip1l | Pericyte |  |  |


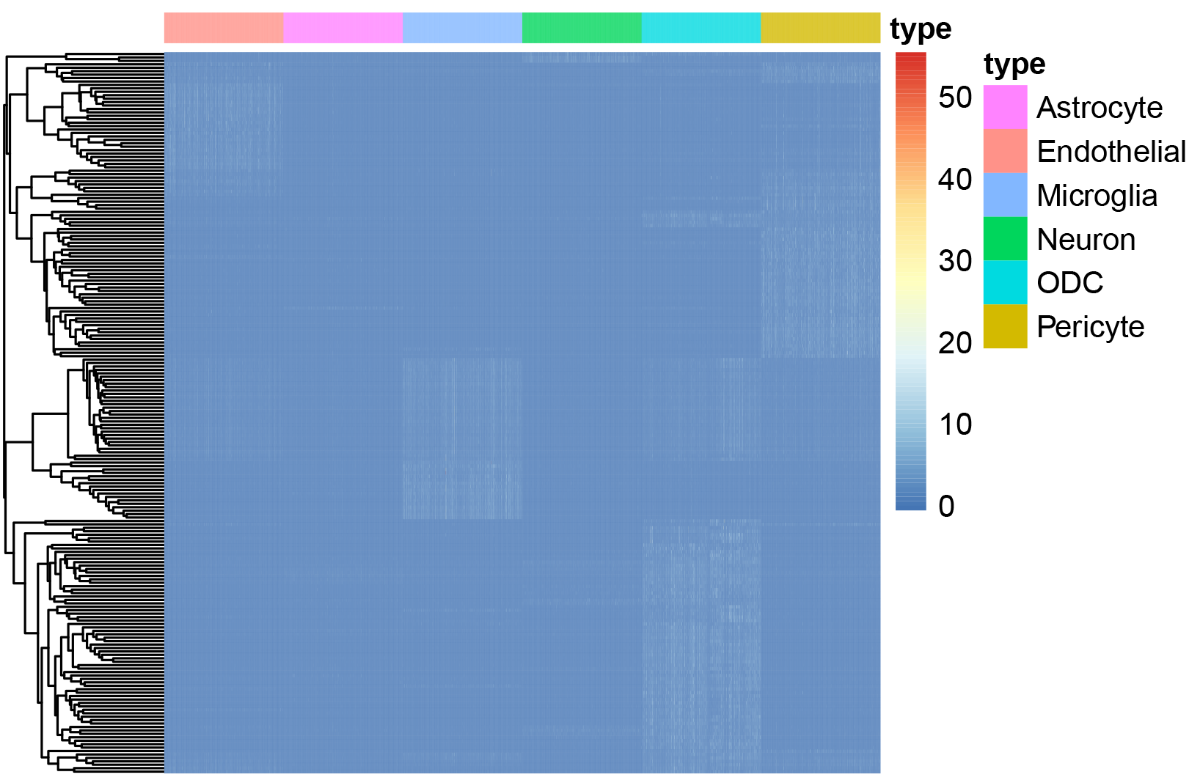


Figure S1 Cluster heatmap of 6000 single cells under ScnML gene set.


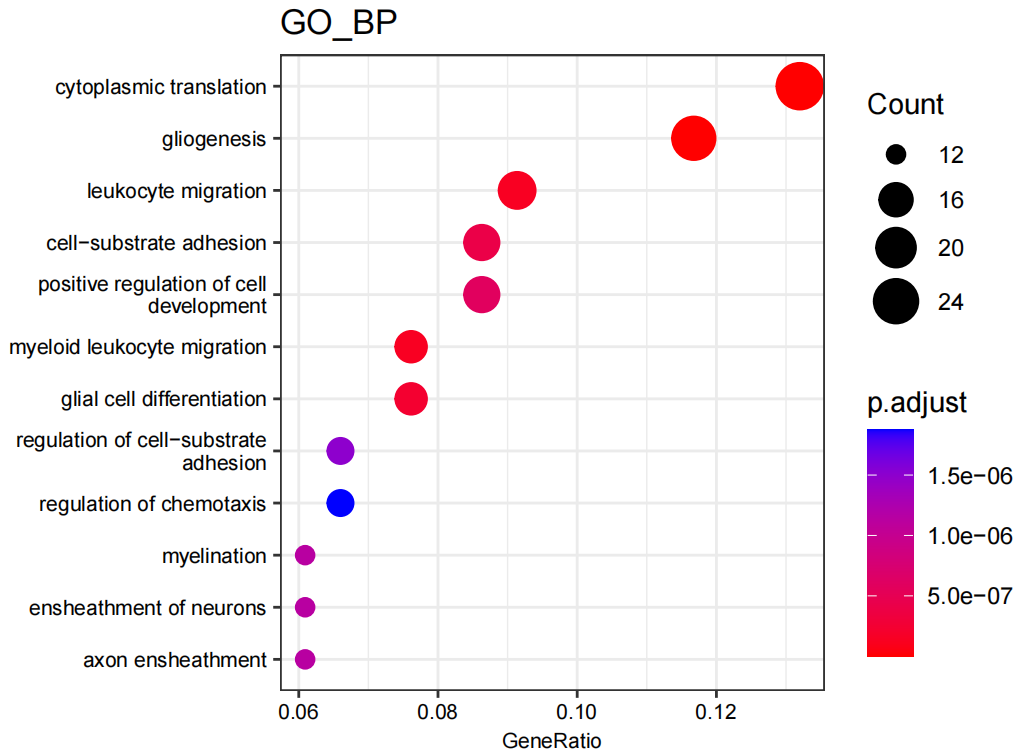


Figure S2 Go analysis of ScnML optimal gene set (biological process).


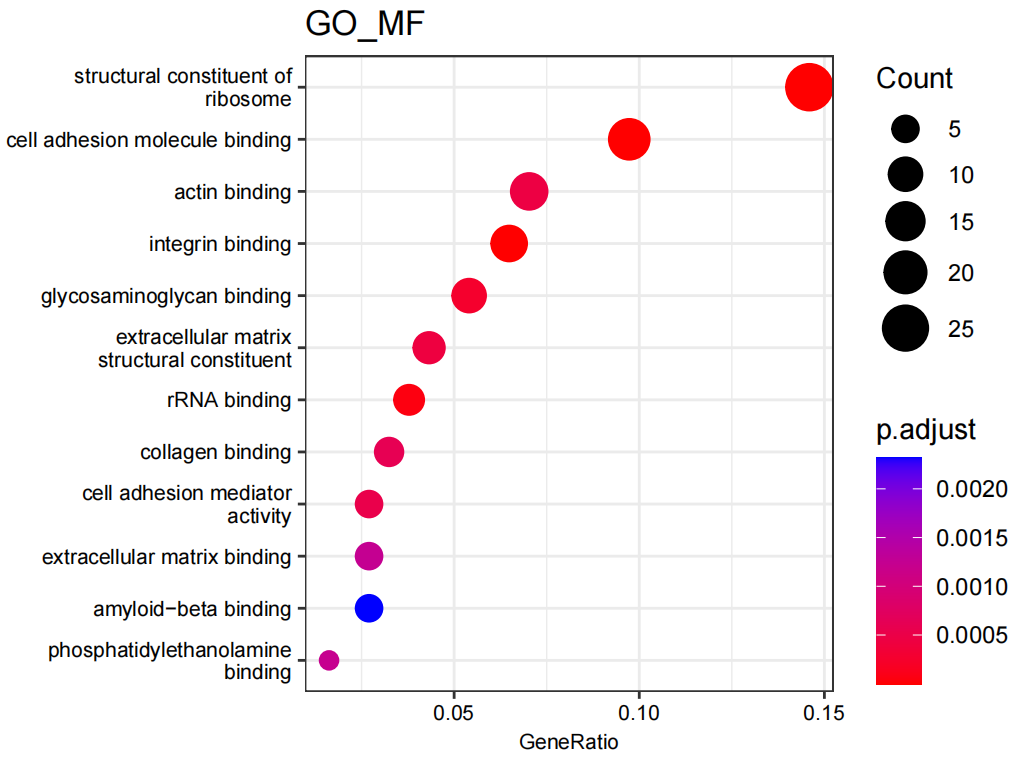


Figure S3 Go analysis of ScnML optimal gene set (molecular function).


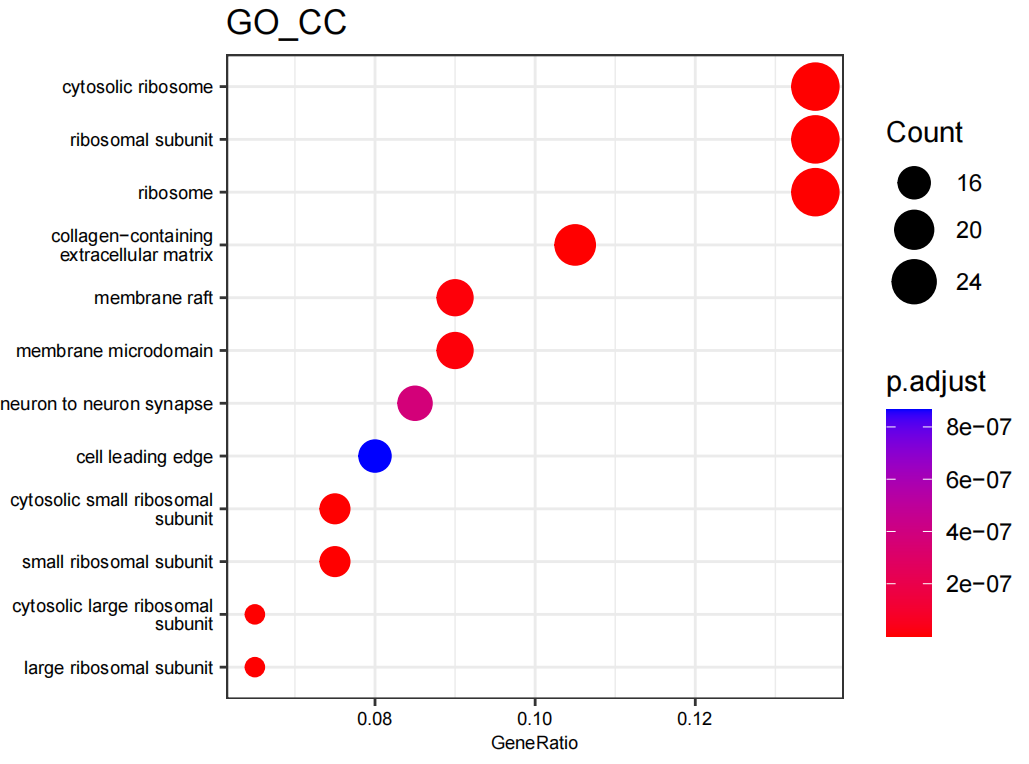


Figure S4 Go analysis of ScnML optimal gene set (cellular component).
