# Supplementary material for: ScnML models single-cell transcriptome to predict spinal cord neuronal cell status
Source: Front Genet. 2024 Jun 4;15:1413484. doi: 10.3389/fgene.2024.1413484 (PMC11183327; doi:10.3389/fgene.2024.1413484)
Supplement: Supplementary file 2 [file Table3.DOCX]

**Original dataset**

Due to file upload size limitations, we have uploaded the original dataset as well as the code to figshare (https://figshare.com/articles/dataset/original_data_zip/25592085). This link is publicly accessible.
